# Development of a textile based protein sensor for monitoring the healing progress of a wound

**DOI:** 10.1038/s41598-022-11982-3

**Published:** 2022-05-13

**Authors:** Yomna ElSaboni, John A. Hunt, Jessica Stanley, Christine Moffatt, Yang Wei

**Affiliations:** 1 Smart Wearable Research group, Department of Engineering, School of Science and Technology, Nottingham Trent University, Nottingham, NG11 8NS UK; 2 Medical Technologies Innovation Facility, Nottingham Trent University, Nottingham, NG11 8NS UK; 3 College of Biomedical Engineering, China Medical University, Taichung, 40402 Taiwan; 4 Skin Integrity, Institute care excellence, Nottingham University Hospital, Nottingham, NG5 1PB UK

**Keywords:** Biomedical engineering, Diagnostic markers, Electrochemistry

## Abstract

This article focuses on the design and fabrication of flexible textile-based protein sensors to be embedded in wound dressings. Chronic wounds require continuous monitoring to prevent further complications and to determine the best course of treatment in the case of infection. As proteins are essential for the progression of wound healing, they can be used as an indicator of wound status. Through measuring protein concentrations, the sensor can assess and monitor the wound condition continuously as a function of time. The protein sensor consists of electrodes that are directly screen printed using both silver and carbon composite inks on polyester nonwoven fabric which was deliberately selected as this is one of the common backing fabric types currently used in wound dressings. These sensors were experimentally evaluated and compared to each other by using albumin protein solution of pH 7. A comprehensive set of cyclic voltammetry measurements was used to determine the optimal sensor design the measurement of protein in solution. As a result, the best sensor design is comprised of silver conductive tracks but a carbon layer as the working and counter electrodes at the interface zone. This design prevents the formation of silver dioxide and protects the sensor from rapid decay, which allows for the recording of consecutive measurements using the same sensor. The chosen printed protein sensor was able to detect bovine serum albumin at concentrations ranging from 30 to 0.3 mg/mL with a sensitivity of $0.0026 \mu$A/M. Further testing was performed to assess the sensor’s ability to identify BSA from other interferential substances usually present in wound fluids and the results show that it can be distinguishable.

## Introduction

Skin is a crucial organ of the human body as it acts as a barrier to protect the rest of the body’s tissues and organs^1^, therefore when it suffers an injury, other essential and healthy organs could become infected or injured^2,3^. While in most minor wound cases, minimal intervention is required such as placing a bandage or medical gauze to prevent further damage to the wound and to prevent it being overrun by infectious microorganisms. However, many chronic wounds need to be monitored and retreated constantly over long periods of time. The cost of treating wounds is a critical issue as it is estimated to account for at least $3\%$ of the total healthcare expenditure in most developed countries^4^. Since 2018, it is estimated that the UK is managing approximately 3.8 million patients with a wound in a clinical setting annually^5^. It was estimated that health services in 2012 spent $\pounds$5.1 billion on costs associated with wound care management^6^ which provided a compelling case for improvement in the current standard of wound dressings not only to reduce healthcare costs but also to improve patient quality of life^7^. However, better and effective means of reporting quantitative information about the wound condition in real time is required to inform and guide treatment decisions as improved wound care will deliver improved public health and healthcare costs^8,9^.

The wound healing process can be monitored by repeatedly determining the multiple physiological changes that occur including but not limited to pH, alkalization, temperature, uric acid and specific protein types such as albumin and fibrinogen whilst tissue repair progresses^2^. Detection of these biomarkers with minimally invasive techniques can provide an effective way for the real-time monitoring of the condition of a wound. In addition, remote wound monitoring could keep the patient informed about their condition, improve their quality of life and reduce the frequency of face-to-face consultations and treatments with healthcare providers^10^.

Higher precision in wound detection treatment is advancing more rapidly as presented recently^3^ where an integrated wound recognition strategy is conducted by extracting patterns of specific irregular wounds. This work was implemented on a bandage, but others have investigated different textiles to allow flexibility of wearable medical devices^11–13^. Integrating electronics with textiles has advanced medical care by facilitating multiple physiological parameters and the body’s biomolecular state to be monitored remotely through minimal or noninvasive techniques and sometimes with reduced direct contact with the human body^13,14^. Improved flexibility and durability allow electronic devices to be more suitable for wearable biosensors as they can be embedded into clothing to realize electronic textiles^15^. These emerging technologies in wearable electronics have made using smart textiles in the design of flexible patches using textiles^11^ which made wound dressings more achievable and is leading to major advances in healthcare monitoring, personalized therapy, and human-machine interaction^12^. The aim of wearable textile biosensors is to indirectly detect critical physiological changes in the body through measuring indirect stimuli that can be readily detected from outside the body despite the uncontrolled environment surrounding the body^16^. However, to be embedded in wound dressings, not only does it have to be miniaturized but it also needs to be flexible and lightweight to make positioning around the wound feasible and as comfortable to wear as possible regardless of the location of the wound^17^. To provide better flexibility the ink used in fabricating the biosensor plays a critical role as adding a bendable layer to a textile can provide fabric reinforcement and make it mechanically bendable^18^.This is one of the challenges that were addressed in this paper and will be discussed in the later section.

The design of the protein sensor is based on the structure of an electrochemical cell that uses a three-electrode configuration to perform the cyclic voltammetry measurements^19–21^. Electrochemical sensors are generally preferred because they can provide rapid real time monitoring of change and wound conditions, they are also relatively inexpensive and can be miniaturized and embedded within textiles^22^. Categorically the device should be a potentiometric biosensor which works by measuring the voltage produced when electric current flows through the solution under static conditions^23–28^.

Screen printing technology has been used to fabricate electrochemical electrodes on ceramic or plastic based substrates^29–31^.This technique involves forcing suitable ink formulations in paste form through a patterned stencil or screened mesh of a specific size and shaped using a squeegee to form the desired design on a substrate^32^. Screen printing provides greater design freedom in that the printed layers can have any orientation on the fabric and do not need to follow the yarn directions. In addition, screen printing provides the ability to produce arrays of the same or different devices in a straightforward fashion; screen printing is inherently a batch process producing multiple devices from a single screen design.

While pH and temperature have been used as parameters in assessing wound status^33–35^, detecting protein concentrations helps to identify wound healing stages^36^ as it is less likely to be affected by the active external environment surrounding the exudate. Albumin was the protein determined in this research work, which has been modelled and measured previously^37,38^ as it is the most abundant protein in blood plasma (it represents $50\%$ of total protein)^39^, previous research has shown a relation between the wellness of a person and the albumin concentration^40^, establishing albumin concentration as a good marker of protein concentrations in wounds. Albumin concentrations in wounds have been used as indicator of wound severity conditions, for which a concentration of $> 15$ mg/mL is in inflamed wounds^41^. While the protein level in a healing wound is around 9 mg/mL, compared to levels of 35 mg/mL for chronic slow healing wounds^42^.

Bovine serum albumin (BSA) was used to prepare standard solutions of albumin^43^. The use of BSA as a protein source specifically when testing electro-chemical sensors has been previously reported by others^44^ because the concentration can be easily standardized and altered precisely to evaluate the detection range. Most importantly, the properties of BSA are very similar to human serum albumin with respect to other proteins as discussed in a recent paper^38^.

For the first time in literature, this research presents a unique approach to integrate protein sensors in fabric which improves the sensors durability, comfort, flexibility and wearability and performance within specification. Although a similar approach was presented recently^8,45^, where screen printed electrodes (SPEs) were printed on a paper and placed inside a bandage but measure pH and uric acid to monitor chronic wounds.

In this research, three designs were investigated to determine the optimal biosensor design for integration into wound dressings. These designs were fabricated by screen printing. Each design was printed directly on 3 different types of fabric with different surface roughness and layer thickness. The conductive tracks were made from silver flake-based ink and carbon ink. UV curable dielectric ink was used to smooth out the textile surface and to provide an even surface for further printing. The same UV ink was used as the encapsulation. 2-point resistance measurement was conducted on printed layers to ensure continuity and cyclic voltammetry measurements were performed to determine sensitivity and selectivity.

## Results and discussion

The research had two main stages. The first stage encompassed the design and fabrication of the textile based sensors and the second stage covered the testing of the sensors using a previously established empirical technique reported by the authors^37^.

### Fabrication of textile based screen printed electrodes

**Figure 1:**
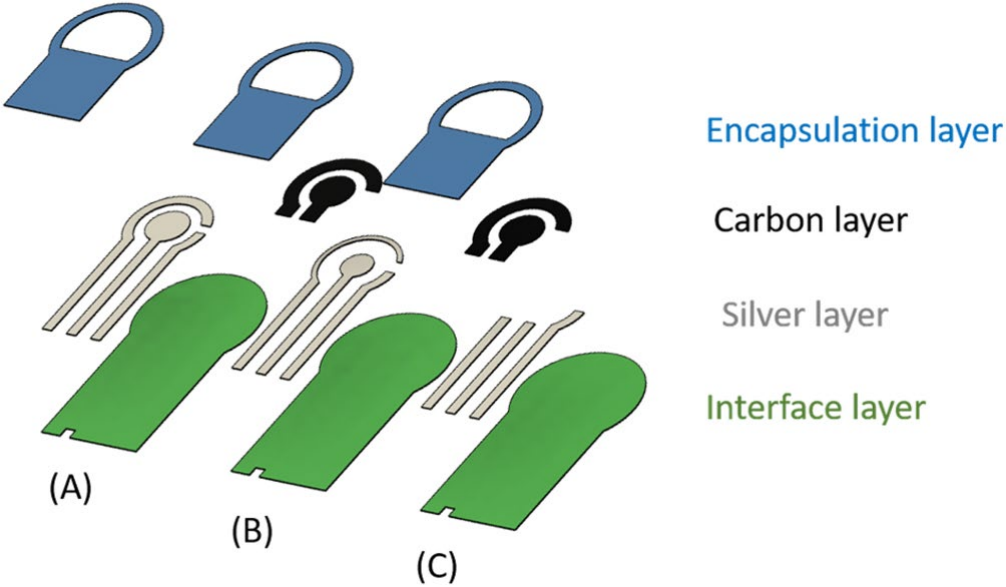
Three SPE designs.

**Figure 2:**
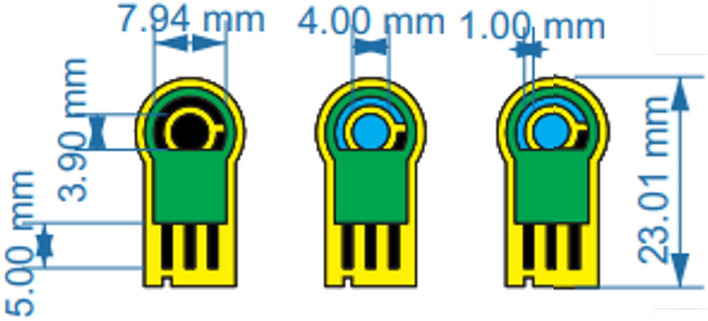
Dimensions of three sensor designs.

The process of screen printing on fabric involves four stages as illustrated in Fig. 1 which shows an exploded view of the three designs. The first design purely consisted of silver layer as both the conductive tracks and electrodes; the second consisted of a mix of both silver and carbon layers; the third design had a carbon layer as part of the electrodes in the region where the sensor encounters the fluid under test at the interface zone. In all three designs, there were three electrodes (working, counter, reference electrodes). The dimensions of the sensors are shown in Fig. 2.

**Figure 3:**
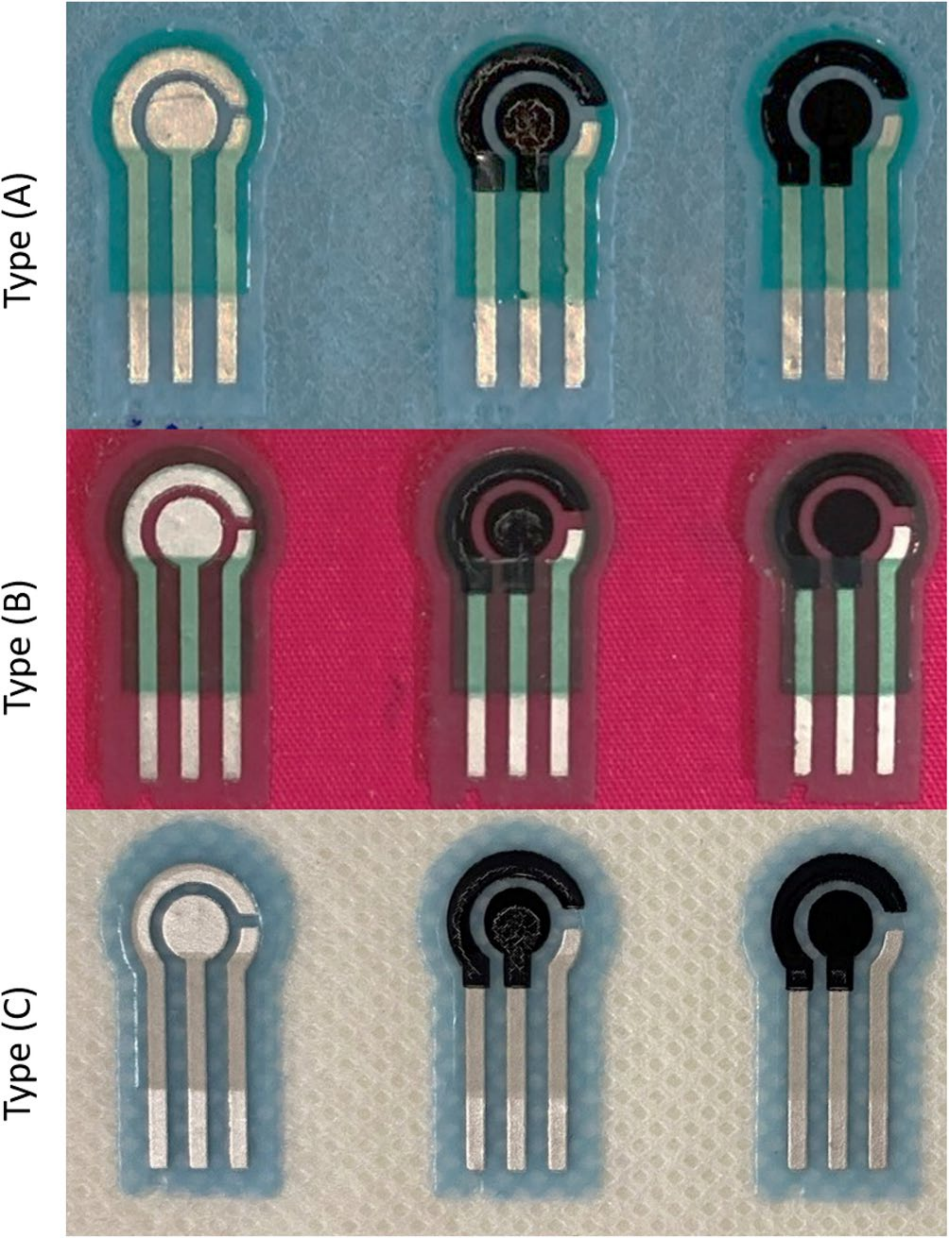
Images of SPEs on the three types of fabric.

The sensors were printed onto three textiles, two of which are medical fabric types. The first one (Type A) was a polypropylene non-woven fabric and has areal surface roughness (Sa = $~119.24$ µm), the second (Type B) is a blend of cotton/polyester woven fabric and has areal surface roughness (Sa = $~59.37$ µm) while the third one (Type C) is made from polyester non-woven fabric and has areal surface roughness (Sa = $~151.52$ µm). Each fabric was placed and adhered in turn onto an alumina tile which provided a rigid platform for printing. The sensors were successfully printed on all three fabric types but it is discovered that printing on the cotton/polyester fabric was easier, while Type A fabric was more difficult to print on because it started to tear apart. However, using polyester non-woven fabric (Type C), which is similar to the one used in wound dressings, prevented the initial substrate layer from cracking. Upon fabrication, the final printed sensors are shown in Fig. 3. After printing, the resistance between two end points of printed track was measured using a multimeter. The resistance observed in design A and B (shown in Fig. 1) was less than 1 ohm on average, while design C always had a much higher resistance (in the range of 260–410 ohms). Microscopic images of the three designs are shown in Fig. 1 in that the tracks were well defined.

The main challenge in the printing process was to maintain the correct alignment when printing each layer to prevent short circuit and to preserve the sensor design. To resolve this issue, a trial print was deposited on a transparent laminate sheet before every deposit to make sure the patterns were all perfectly aligned before printing directly on fabric. The second main challenge faced repeatedly was the roughness of each printed layer. To print several layers on top of each other, all layers need to be smooth with no pin holes on the surface as this affected the roughness of the final finishing layer. To address this issue, the smooth side of the fabric was initially shaved to enable the surface to be as uniform as possible. The dielectric layer was then printed several times with varying printing gaps to reduce any pin holes and to provide an even platform for printing conductive layers. The third challenge was that the fabric could not be easily removed from the alumina (supporting platform). It was observed that this only occurred when using thin fabric such as Type A fabric shown in Fig. 3. To avoid this issue, two layers of the same fabric were adhered and fixed on top of each other. This strengthened the fabric layer but also eased the removal of fabric after printing.

**Figure 4:**
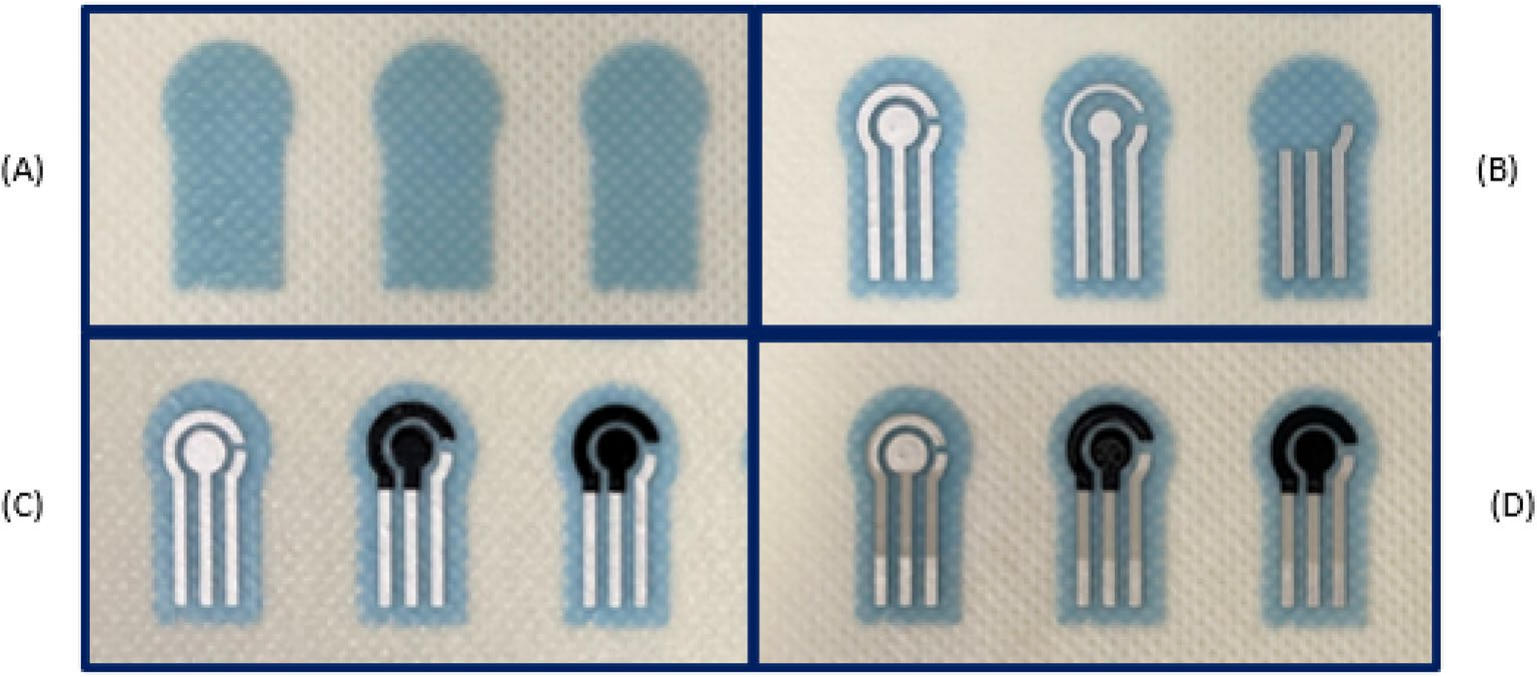
Different stages of screen printed electrodes (**A**) interface layer, (**B**) Silver, (**C**) Carbon, (**D**) Encapsulation.

**Figure 5:**
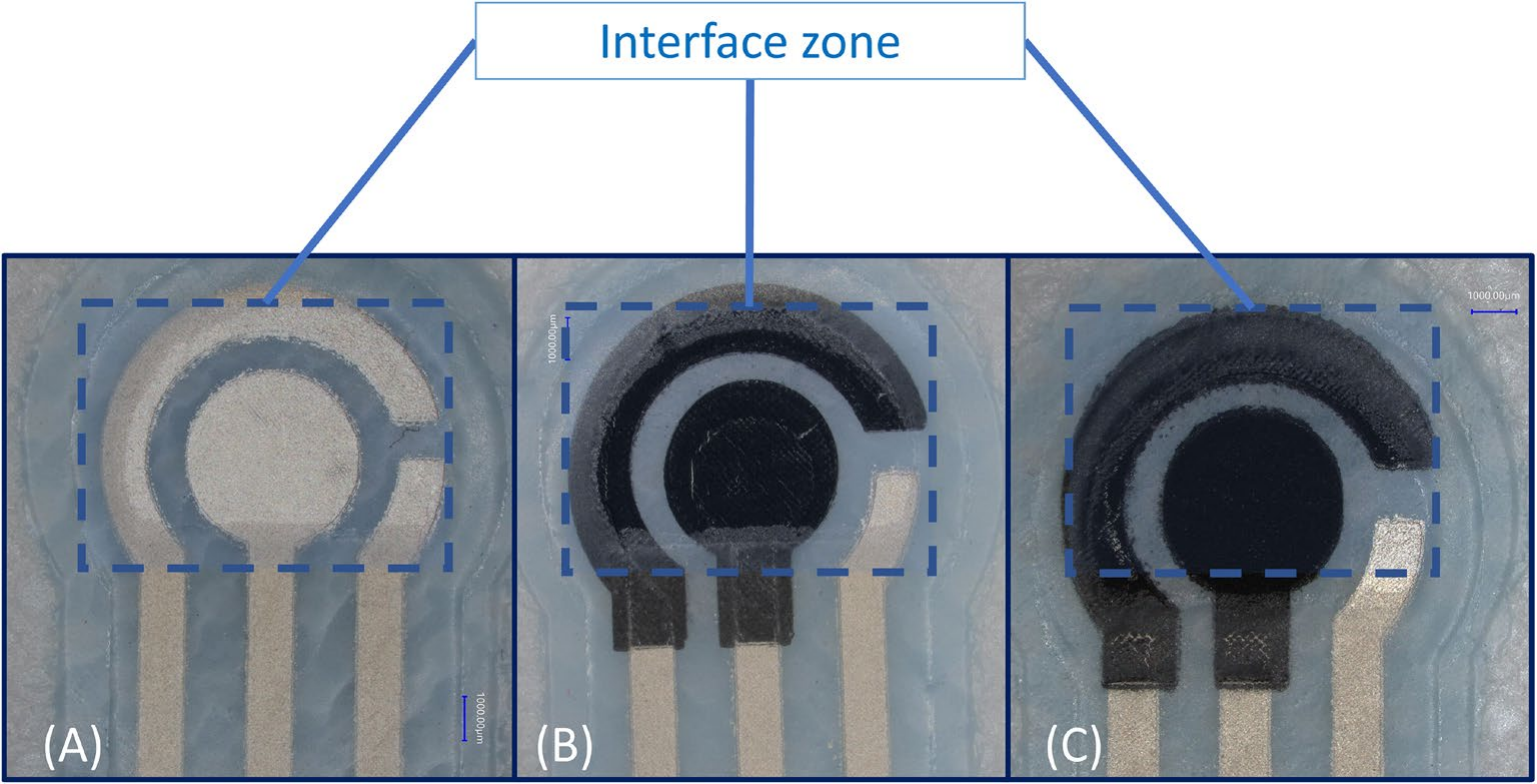
Microscopic images of the three textile SPEs. (**A**) Silver only SPE, (**B**) Carbon and silver SPE and (**C**) Carbon only SPE.

The printed layers after each stage are shown in Fig. 4. Initially, in the first stage shown in (A), the polymer interface layer was deposited six times to create a smooth platform for subsequent layers to be printed on top. The interface layer was then cured under UV light to produce a thickness of $~110$ µm. Next, the silver conductive electrodes were printed as shown in (B) and cured in the oven for 15 minutes at 100 $^{\circ }$C. The silver layer was printed twice to produce a thickness of $~16$ µm. The carbon layer was then printed only on the second and third designs as shown in Fig. 4C and cured in the oven for 15 minutes at 100 $^{\circ }$C. The print was repeated twice to create a thickness of $~26$ µm. Finally, the same material used for the interface layer was printed to protect the conductive tracks of the sensors as shown in (D) and was cured under UV light to produce a thickness of $~14$ µm. A microscopic image of the interface for each design before being tested is shown in Fig. 5. The images captured show how the electrodes in delicate area of the interface zone are clearly separated from each other and how the layers are well aligned and printed on top of each other and there is no short circuit occurring.

**Figure 6:**
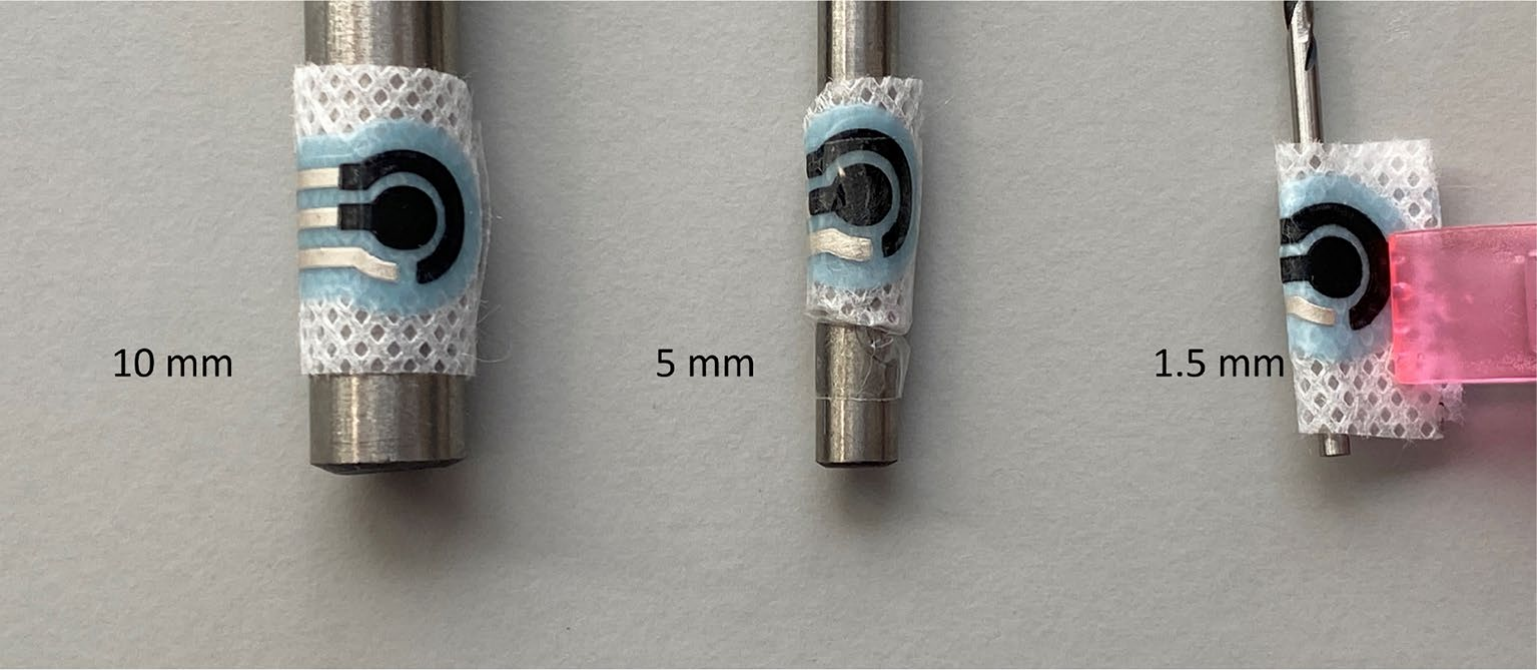
Flexibility test of printed sensors by wrapping around cylinders with a diameter ranging from 1 to 10 mm (only 10 mm, 5 mm and 1.5 mm are shown above).

**Figure 7:**
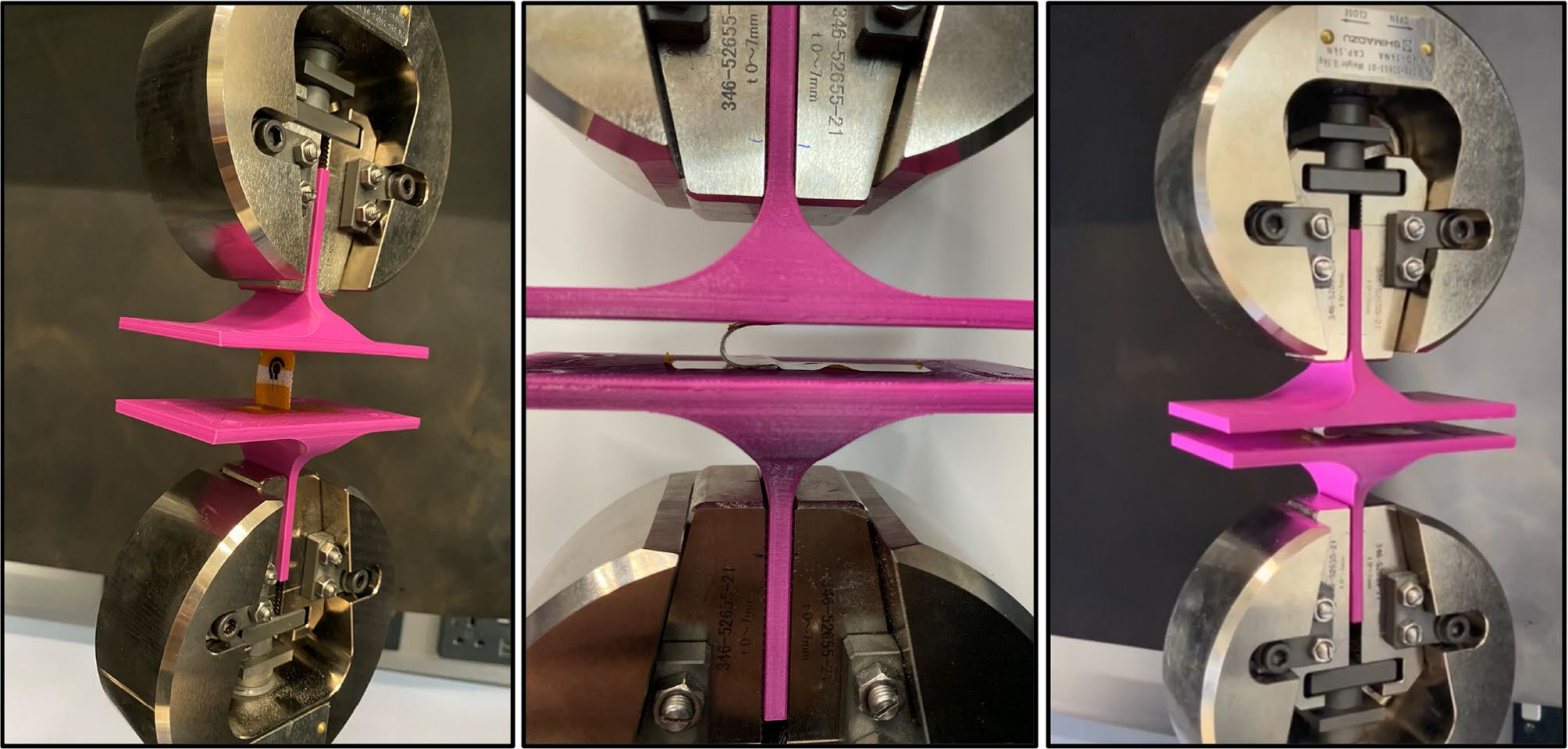
Shimadzu mechanical bending setup with bespoke attachments.

Upon fabrication, the sensors were highly flexible and can be easily bent as shown in Fig. 6, where the sensor was wrapped around cylinders with a diameter ranging from 1 mm to 10 mm to mimic the bending when worn on patient. The results showed that all designs could wrap around these diameters without causing cracks in any of printed layers. This was further cross validated through continuity measurement, proving the conductivity of printed sensors is intact. This feature is of great significance because of the nature of the targeted application as the exact positioning of a wound is unpredictable, and the dressing does need to be wrapped and bent around the human body. To test the bendability of the sensor, an experimental setup was prepared as shown in Fig. 7. This was achieved using a Shimadzu AG-X Universal Testing Machine with custom 3D printed attachments to grip the printed sensors. The two attachments were initially set at 21mm apart which is the maximum distance from two opposite sides of the sensor. These attachments were them moved towards each other and stopped at mm apart to avoid damage to machine and attachment. The cyclic test was repeated 5 times with 3 sensors. The measured force to bend the sensor ranges from 0 to 2.5 N depending on compression position and there was no damage to the integrity of all designs which was subsequently cross-validated through continuity measurement. This mechanical test demonstrated a quantifiable measure of the sensor ability to bend with minimal force without affecting the sensor’s structure. After the test was conducted, the conductivity of the electrodes was measured and remained unchanged and unaffected by the test.

### Cyclic voltammetry (CV) measurements

**Figure 8:**
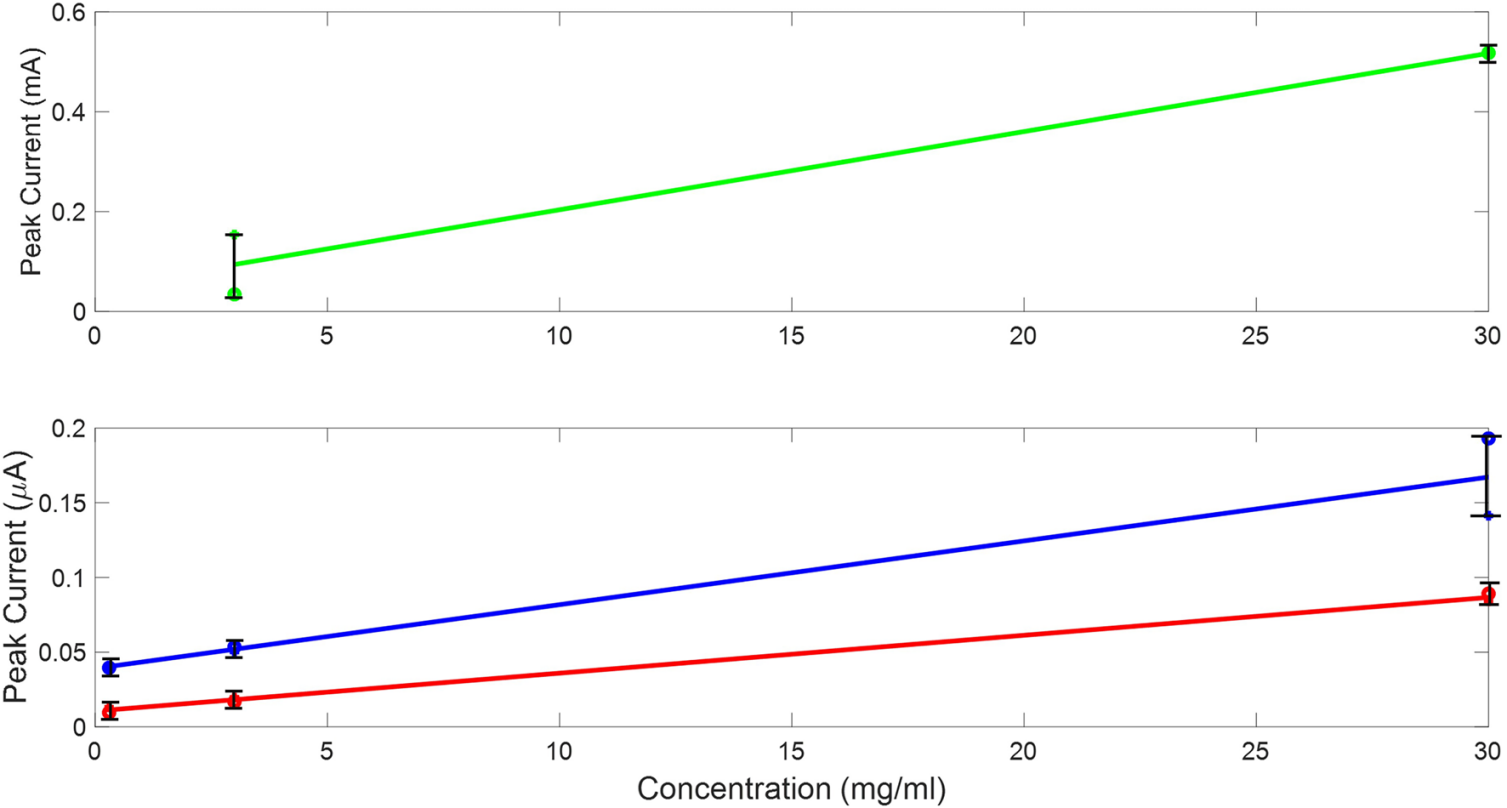
The oxidation peak observed after repeating the experiment twice using three designs (top) Design A, (Bottom) in blue is design B and in red design C.

The fabric around the bottom edge of the sensor was removed in order to be connected to the AUTOLAB Dropsens adaptor which includes an insertion connector with 3 pins. BSA with 8 concentrations ranging from 0.3 - 30 mg/mL were used as the protein sources. Initially, the experiment was conducted on three sensor designs on all three types of fabric to establish the most suitable design. For each measurement, the current at the oxidation peak was observed and recorded and a best fit line of all the measurements was then plotted in Fig. 8. The SPE design with only silver layer at the interface zone (SPE design (A)) stopped functioning when testing a solution with concentration below 3 mg/mL. As shown in Fig. 8, the two cycles conducted using SPE with only carbon were close to each other in terms of gradient as there was less than $8\%$ difference in comparison with design A and B where the differences in measurements between two cycles were $32\%$ and $41\%$, respectively. This demonstrated that design C was the most stable and reliable design as it provided reproducible and comparable results each time. The results show a clear relationship between BSA concentration and current peak obtained at the working electrode. This happens because the increase in concentration gradient near the surface of the working electrode causes an increase in the current observed at the oxidation peak. This is a result of the increase in the amount of electroactive proteins adsorption at the interface when the concentration of BSA increases, leading to increase in surface charge density which drives higher current at the oxidation peak^46^.

**Figure 9:**
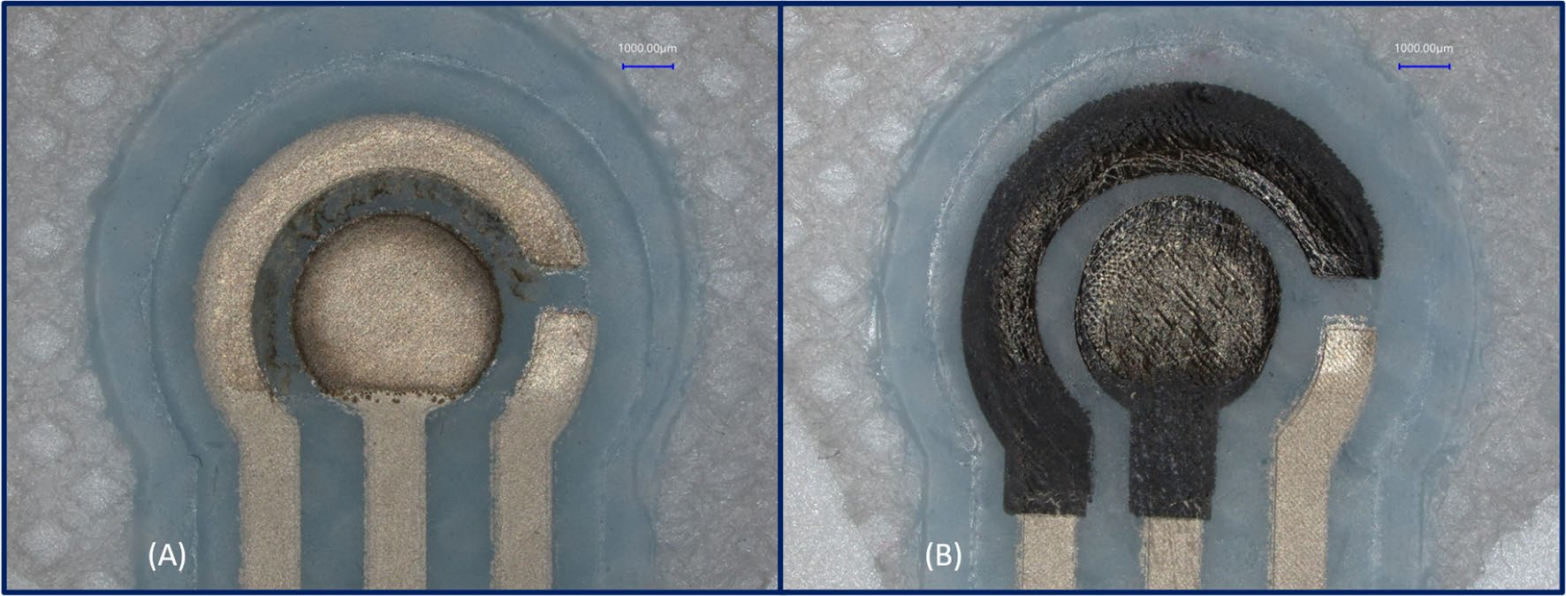
The silver containing SPEs at the interface zone after use (**A**) silver only based SPE and (**B**) Silver and Carbon SPE.

**Figure 10:**
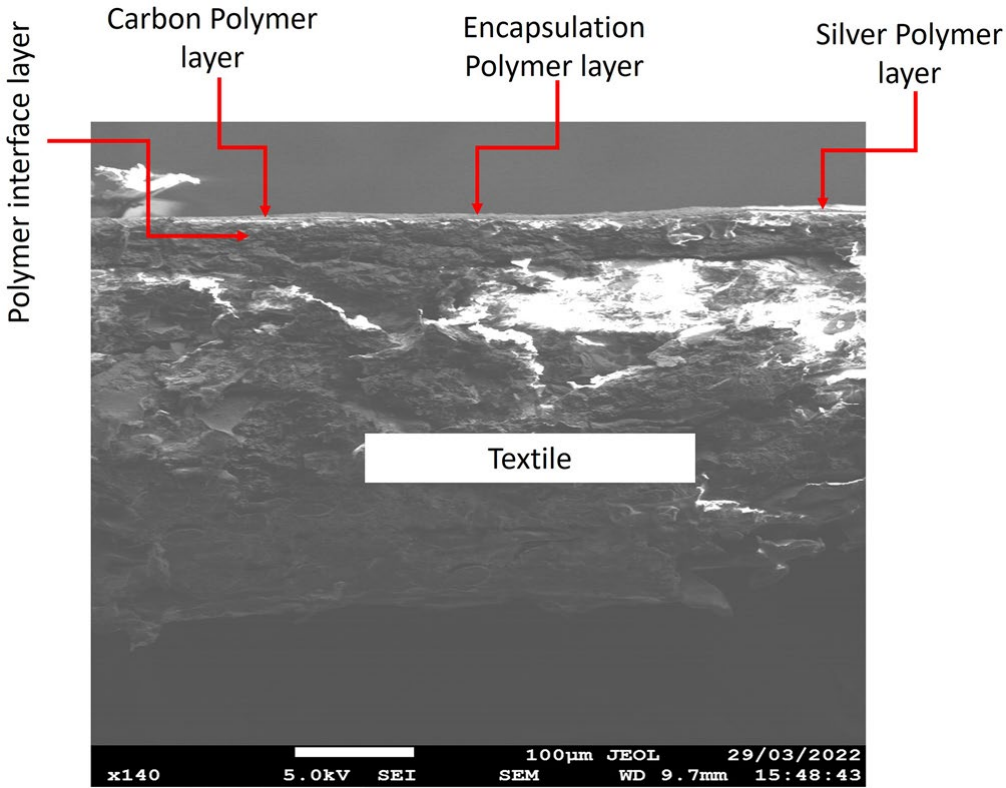
A cross-sectional SEM micrograph of SPE Design (C) around interface zone.

The status of the sensors after CV measurement performed was investigated to ensure no printed material was damaged. This was achieved by examining each sensor post measurement under the microscope. During the measurement process, the formation of a dark grey color coating (Silver dioxide) was observed at the working electrode when using SPE design (A) as shown in Fig. 9A. This indicated that the silver electrode was damaged before the end of the cycle and could lead to unreliable results. In order to examine the underlying silver layer in design B, the carbon layer was manually removed to expose the silver layer. SPE design (B) where the carbon layer was scratched in SPE design (B) to make the silver layer more visible as shown in Fig. 9B. The image illustrates how silver layer was still oxidized, but was far less visibly damaged than design (A) because it is protected by the carbon layer as it is noncorrosive. The SPE design (C) remained unchanged as there was no silver present at the interface zone. SPE design (C) was then observed using a scanning electron microscope (SEM) and a cross-sectional micrograph including the conductive tracks, the encapsulation layer, interface layer and the textile is shown in Fig. 10. The image shown demonstrates the continuity and consistency of the multiple layers present in design of the sensor around the interface zone.

**Figure 11:**
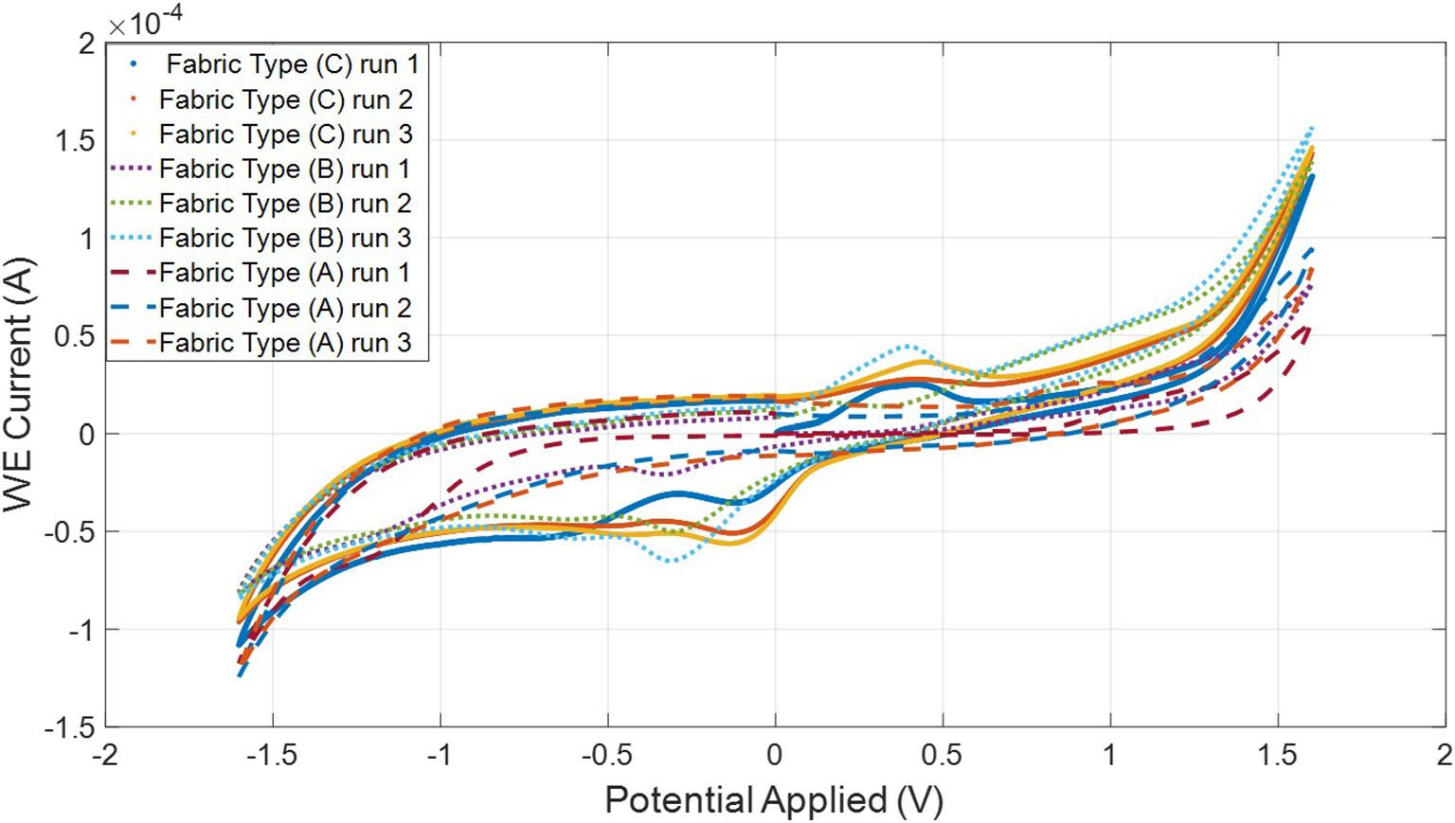
Measurements obtained when testing 30 mg/mL of BSA solutions on SPE design (C) and conducting three cycles on the three types of fabric.

Further empirical testing was only conducted using SPE design (C). The CV measurement obtained after the redox reaction is shown in Fig. 11 when 30 mg/mL BSA was used. After repeating each test three time with new sensor on each fabric type, there was a correlation observed after the first cycle between cycle 2 and 3. The equation of the line for the sensors with three different fabric types are included in Table 1.

**Table 1:** The relationship between number of cycles and CV measurements.

Fabric type	Equation	Cycle 1
Fabric type C	y = $0.3*10^-5 x + 10$ Cycle 1	$0.13*10^-3$
Fabric type B	y = $0.186*10^-4 x + 13$ Cycle 1	$0.77*10^-4$
Fabric type A	y = $$0.112*10^-4 x $$+ Cycle 1	$$0.54*10^-4$$

**Figure 12:**
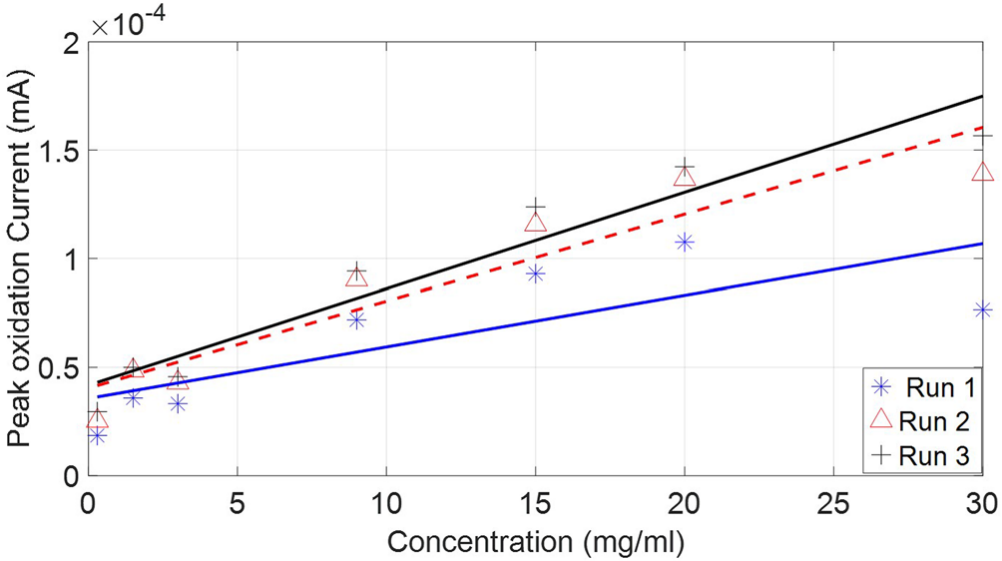
Measurements obtained when testing 8 BSA concentrations on SPE design (C) and conducting 3 cycles for each sample on each sensor.

The CV experiment was then repeated three times on the same sensor and performed on all three types of fabric. The results obtained using the three types of fabric overlapped each other and were within the same current range at the working electrode (0.06–0.15A). Therefore, the effect of fabric type on the redox reaction was minimal. Type (C) fabric was more repeatable as clearly shown in the graph (the undashed lines) and was chosen as the standard in the fabrication of the sensor because it is also commonly used for medical wound dressings. In addition, the first CV cycle was always lower in magnitude than the measurements obtained in the subsequent cycles. The second and third cycles were consistently closer to each other in comparison with the initial cycle. This is more visible when all peaks of several concentrations of each cycle were compared to each other as shown in Fig. 12. Therefore, as a standard it is best to take the average of the second and third cycles when comparing the results.

Since the combination of type (C) fabric and SPE design (C) was ideal, eight BSA concentrations were used to examine the sensitivity of this combination. The current observed at the oxidation peaks were recorded, shown in Fig. 12. The best fit line was drawn based on the average value around each solution of each cycle. While the initial cycle was consistently lower in gradient as presented in Table 2 than the second and third cycles conducted on the same un-replaced sensor which illustrates that SPE design (C) is the most reproducible.

**Figure 13:**
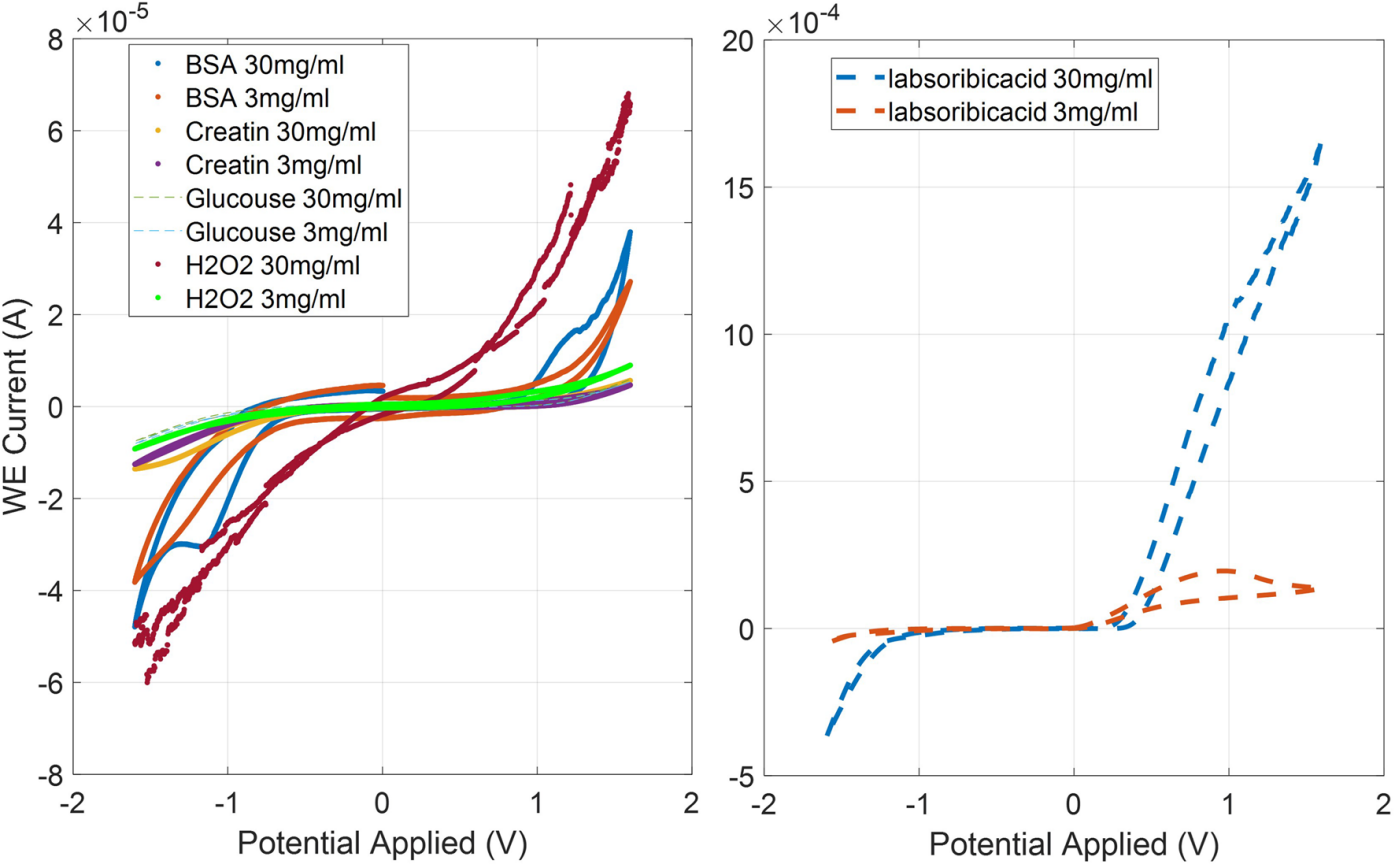
Comparison between potential interferential substances using combination of type (C) fabric and SPE design (C).

After testing the sensitivity of the chosen SPE sensor, it was important to analyse the selectivity of printed sensor in comparison with other substances present in wounds. Therefore, the SPE was tested against several potentially interfering substances such as creatin, hydrogen peroxide, glucose and absorbic acid and the results are shown in Fig. 13 similar to a study achieved previously^47^. In Fig. 13 the shape and range of the redox reaction obtained through CV of BSA is different from the other tested solutions tested which eliminates the effect of interference.

**Table 2:** The best fit line of oxidation peak measurements with all three designs using type (C) fabric.

SPE Type	Sensitivity ($\mu$**A/M**)	zero-point ($\mu$**A**)
Design (A) Trial 1	18	10
Design (A) Trial 2	13	120
Design (B) Trial 1	0.0034	0.04
Design (B) Trial 2	0.0052	0.038
Design (C) Trial 1	0.0024	0.012
Design (C) Trial 2	0.0026	0.009

## Conclusion

The sensor was fabricated using the screen printing technique and consisted of four layers: an interface layer, silver electrodes, carbon conducting interface area and an encapsulation layer. The sensor relied on quantifying the protein concentration by measuring changes to conducting cyclic voltammetry measurements. After testing three sensor designs with different silver and carbon combinations, it was concluded that that the design with only carbon material presented at the interface zone was the optimal design as not only it provided the most reproducible and consistent results upon testing, but also it remained unaffected by oxidation. The sensor was printed on three different fabric types, and all presented promising results, one of which was wound dressing fabric which was chosen as the substrate as it is commonly used medically. During the screen printing process, precise alignment is a key aspect in the fabrication process to prevent any short circuit of the electrodes and to provide efficient performance of the sensors. Upon further cyclic voltammetry empirical testing and result analysis conducted using carbon only sensors (design (C)) and on Type C fabric, it was determined that to compare the outcome of different BSA concentrations it is best to take the average of the second and third cycles conducted on the same sensor. The CV measurements demonstrated that the screen printed protein sensor could accurately monitor BSA concentrations from 0.3 to 30 mg/mL with a sensitivity of $0.0026\, \mu$A/M. Additionally, an SEM image was captured and presented in the paper to demonstrate the consistency and continuity of the different layers making up the texture of the final fabricated design. The final design also demonstrated its ability to bend easily around different diameters without breaking. Further testing was conducted to assess the sensor’s ability to detect BSA from other interferential substances present in a wound. The measured results show that the chosen SPE type (C) can successfully distinguishes BSA in the range of 3 to 30 mg/mL from others.

## Methods

### Screen printing process

#### Layer types

To design the protein sensors shown in Fig. 6, the sensor consisted of four printed layers. Initially, a layer was built to create an interface for the electrode conductive tracks. Silver layer was then deposited over the interface layer. In two of the designs (design B and C), carbon conductive ink was deposited over the interface zone. Finally, an encapsulation layer was deposited on top of the sensor tracks and around the interface zone to protect the sensor electrodes and to prevent any short circuit. In this work, three different designs were considered when designing the carbon conductive layer.

#### Ink types

Three types of inks were used: a UV curable polymer ink from ElectraPolymers Ltd was used to act as the interface and encapsulation, carbon and silver inks from Henkel were used as the electrodes and conductive tracks, respectively.

#### Textile materials

Three different fabric types were tested: 1) a non-woven polypropylene fabric (Type A), 2) a woven polyester/cotton fabric ($65\%$/$35\%$) (Type B), 3) a non-woven polyester fabric (Type C). Type A and C are commonly used in used in wound dressings and therefore are more favorable. Although the woven polyester/cotton (Type B) fabric is the most ideal type of fabric for printing because it is relatively smoother than the others (has areal surface roughness (Sa) = $~59.37$ µm), yet it is not commonly used in wound dressings. We only use Type C as a comparison.

### Protein solution preparation

The protein used to test the printed sensors was BSA powder as it is considered as a standard for protein quantification (Sigma-Aldrich, Steinheim, Germany). In this research, 8 samples of BSA solutions were prepared with different concentrations and diluted using deionized water: 30, 23, 18, 11, 7, 3, 1, 0.3 mg/mL with a pH value of 7. Initially, 30 mg/mL was prepared by putting 3 grams of BSA powder in 100 mL of deionized water, and then the rest of the solutions were prepared by diluting the original stock solution.

### Measurement setup

**Figure 14:**
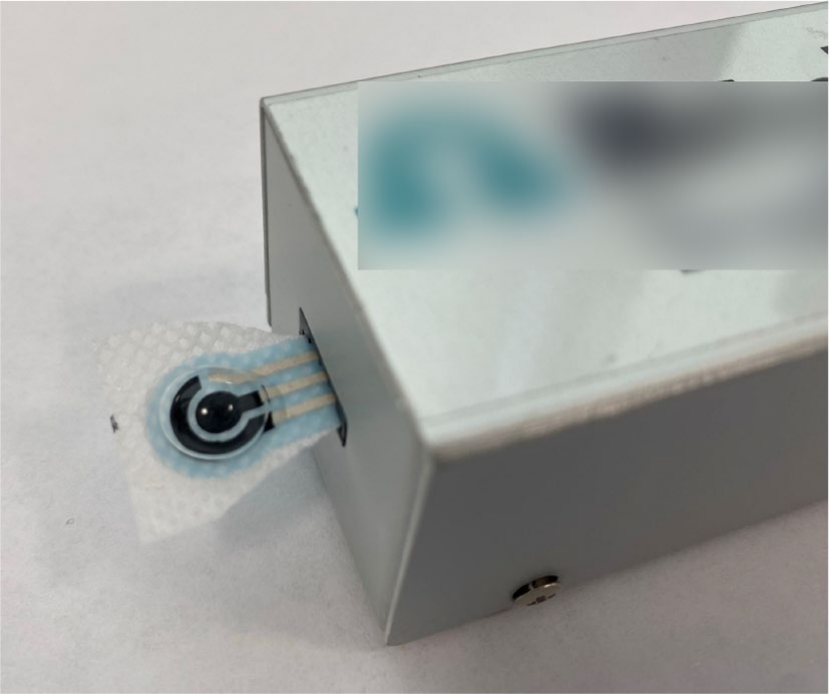
Conducting CV measurements on a BSA sample using SPE Design C connected to an adaptor.

A setup similar to the methodology discussed in the previous work by the authors was implemented. The objective is to evaluate the sensitivity shown in Table 2 of the developed textile-based screen printed carbon electrodes as shown in Fig. 14. The printed protein sensor was connected the Metrohm Dropsens device. A glass filled with ice was placed within a close proximity to the sensors to provide humid environment and to prevent evaporation of the BSA samples. This was achieved by conducting a series of cyclic voltammetry experiments on different protein solutions using an AUTOLAB potentiostat device (PGSTAT101). The setup parameters are listed in Table 3.

**Table 3:** Parameters of cyclic voltammetry measurement setup.

Parameter	Value
Measurement type	CV staircase
Scan rate	0.08 V/s
Start potential	0.0 V
Step	0.0025 V
Upper vertex potential	1.6 V
Lower vertex potential	$-$ .6 V
